# Institutional Notes and News

**Published:** 1910-02-19

**Authors:** 


### Institutional Notes and News.

**Worcester Ophthalmic Hospital.**

At the annual meeting of this hospital the report was presented, showing an increase of income, £563 6s. 3d., in 1909, as against £444 10s. 7d. in 1908. The expenditure had been £537 13s. 11d., as against £414 6s. 4d. The out-patients numbered 1,112, and the in-patients 59. The Chairman, in moving the adoption of the report, called attention to the satisfactory financial position, and to the increased work, which, being largely among children, might to some extent be looked upon as preventive work. He was satisfied that the institution is worked economically, thanks to the unceasing care of the sister in charge.

**Doctors and School Children at Norwich.**

The question of the relation of the hospitals to the educational authorities, resulting from the medical inspection of school children, is very pointedly illustrated in the report of the medical staff of the Norfolk and Norwich Eye Infirmary. Dr. Arthur Greene, who presented it on February 1 at the annual meeting of subscribers, points out, for instance, that the number of out-patients now is nearly double what it was in 1907, and that this increase is due to the large number of school-children who come for treatment as a result of medical inspection. Dr. Greene further went on to say that the honorary medical staff is placed in an unfortunate dilemma, because, while it is naturally unwilling to deprive the children of any services, at the same time, owing allegiance to the British Medical Association, it has to remember that that Association strongly disapproves of honorary medical officers to hospitals being regularly employed by public authorities. Luckily no hasty action is to be taken, so that time may be allowed to work a compromise. For what is the position of the educational authorities with regard to it? The annual report of the chief medical officer of the Board of Education just issued points out the lines on which the difficulty will probably be solved. He says: "From the first the Board has recognised that the use of hospitals in this way is only possible in certain areas, and that in other areas such institutions do not exist, or, though existing, were over-taxed already. Where, however, available and where appropriate, the Board has sanctioned proposals for contributions to the funds of these institutions on terms of adequate advantage, including, where desired, the allocation of reasonable remuneration to the medical staff." Of course, an eye hospital has felt the change more quickly than other institutions, but in the face of the above it is only reasonable to expect a settlement, and for either side to provoke a breach would only harm the most important party, which is not the Board of Education nor the British Medical Association, but the child.

**Harrogate Royal Bath Hospital.**

A very satisfactory annual report, including a credit balance, was read at the recent annual meeting of the governors and subscribers to the Royal Bath Hospital and Convalescent Home, Harrogate. Patients admitted during the year numbered 1,528, as compared with 1,399 in 1908. Of these 1,109 were treated in the hospital, and 419 were admitted to the Home—120 more than last year. This increase, said the report, was certainly due to the alteration of the rules by which the Home patients are admitted on recommendation for two weeks free of further charge, instead of three with payment, an alteration of which over half the patients availed themselves. This increase was likely to continue when the change and its advantages were more fully understood by patients and subscribers. The total expenses for the hospital and home amounted to £2,974, and this amount had been divided according to rule in proportion to the number of patients admitted to the two. During the year 7,861 baths of all kinds had been given, of which the large majority was sulphur-water baths. A year's salary of £90 had been given to the matron, Miss Waters, who was retiring, after thirteen years of good work, owing to an unfortunate trouble with her sight.

**Salford Union Infirmary.**

Reporting to the Salford Guardians, the Clerk said he had written to the Local Government Board stating the Guardians' proposals for the administration of the Hope Hospital, and had received a letter in reply approving of the proposal to appoint a medical superintendent to have sole charge of the institution. This would dispense with the services of the visting medical officer, to whom, in the circumstances, the Board approved the payment of a gratuity of £250. The salary of the medical superintendent will commence at £350, and will rise to £500 a year, with an allowance for rent and taxes.

**Is Chichester Infirmary in Debt?**

The affairs of the West Sussex and East Hants and Chichester Infirmary have been causing so many searchings of heart that the report of a sub-committee to consider the financial position has become a matter of general interest. The fact upon which the report is based is what has been called a debt of £1,500. There is a particular reason just now that every effort should be made to show a clean balance sheet, because the King will consider the question of allowing the hospital the style of "Royal," provided the debt is paid. Apart from the proposal that the infirmary ball shall be revived, bazaars held, and other expedients of the kind resorted to, the committee suggested that subscribers of 5s. would be encouraged if one out-patient's letter were given in return. As the cost of each out-patient is any way not more than 3s., a gain of nearly 50 per cent. would result to the infirmary. Another proposal of a similar kind was that an in-patient's letter should be given for a subscription of £3 3s. instead of for a subscription of £2 2s. as at present. Mr. Edward Hawes, however, attacked the report on the general and substantial ground that as there was a reserve fund of £15,000 absolutely at the disposal of the governors it was ridiculous to say that the infirmary was in debt at all. He said they were in their present position because they objected to put their hands in their pockets to pay what they owed, and that if the reserve fund was not to be drawn upon on an occasion like the present what was the use of having a reserve fund? The Secretary, Mr. Marshall, then admitted that in 1894 power was granted to the governors to sell out to the extent of £1,258, but it had never been exercised, in the hope that something would turn up. Major Jellicorse, however, strongly opposed this proposal, and said that instead of selling out some of the reserve fund, it should rather be raised by five thousand more. The meeting seemed to concur, as Mr. Hawes' motion for selling out was lost. Mr. Marshall said the patients had quadrupled since he was appointed, and, as he was unable to do all the work, it was decided to appoint a paid Secretary, the whole of whose time would be at the service of the institution. So many of the Committee's proposals were negatived that it is difficult to gather what new policy, if any, is to be pursued. The position appears to be much what it was before the meeting.

